# The Catechol-O-Methyltransferase (COMT) val^158^met Polymorphism Affects Brain Responses to Repeated Painful Stimuli

**DOI:** 10.1371/journal.pone.0027764

**Published:** 2011-11-21

**Authors:** Marco L. Loggia, Karin Jensen, Randy L. Gollub, Ajay D. Wasan, Robert R. Edwards, Jian Kong

**Affiliations:** 1 Department of Anesthesiology, Perioperative and Pain Medicine, Brigham and Women's Hospital (BWH), Harvard Medical School (HMS), Boston, Massachusetts, United States of America; 2 Department of Psychiatry, Massachusetts General Hospital (MGH), Harvard Medical School (HMS), Boston, Massachusetts, United States of America; 3 MGH/MIT/HMS Athinoula A. Martinos Center for Biomedical Imaging, Boston, Massachusetts, United States of America; 4 Department of Psychiatry, Brigham and Women's Hospital (BWH), Harvard Medical School (HMS), Boston, Massachusetts, United States of America; Hangzhou Normal University, China

## Abstract

Despite the explosion of interest in the genetic underpinnings of individual differences in pain sensitivity, conflicting findings have emerged for most of the identified “pain genes”. Perhaps the prime example of this inconsistency is represented by catechol-O-methyltransferase (COMT), as its substantial association to pain sensitivity has been reported in various studies, but rejected in several others. In line with findings from behavioral studies, we hypothesized that the effect of COMT on pain processing would become apparent only when the pain system was adequately challenged (i.e., after repeated pain stimulation). In the present study, we used functional Magnetic Resonance Imaging (fMRI) to investigate the brain response to heat pain stimuli in 54 subjects genotyped for the common COMT val158met polymorphism (val/val = n 22, val/met = n 20, met/met = n 12). Met/met subjects exhibited stronger pain-related fMRI signals than val/val in several brain structures, including the periaqueductal gray matter, lingual gyrus, cerebellum, hippocampal formation and precuneus. These effects were observed only for high intensity pain stimuli after repeated administration. In spite of our relatively small sample size, our results suggest that COMT appears to affect pain processing. Our data demonstrate that the effect of COMT on pain processing can be detected in presence of 1) a sufficiently robust challenge to the pain system to detect a genotype effect, and/or 2) the recruitment of pain-dampening compensatory mechanisms by the putatively more pain sensitive met homozygotes. These findings may help explain the inconsistencies in reported findings of the impact of COMT in pain regulation.

## Introduction

Sensitivity to pain varies greatly across humans and growing evidence suggests that genetic factors might explain part of this variability [1]–[8]. Among the few single nucleotide polymorphisms (SNPs) that have been suggested to be associated with pain, one that has recently attracted significant attention is Catechol-O-methyltransferase (COMT) val^158^met. COMT is an enzyme that is involved in a number of physiological functions, including the degradation of catecholamine neurotransmitters after their release in the synaptic cleft [9], [10]. The val^108/158^met SNP is associated with a valine(val)-to-methionine(met) substitution at position 108 or 158, which leads to a four-fold decrease in enzyme activity in met homozygotes, with the heterozygotes demonstrating intermediate activity [11], [12]. The first direct evidence that this polymorphism affects neural processing of pain came from Zubieta and colleagues [8], who showed that ^158^met homozygotes were characterized by higher pain sensitivity, diminished regional mu-opioid system responses to pain, as well as a higher mu-opioid receptor binding potential, compared with heterozygotes (and vice versa for the subjects ^158^val homozygotes). Despite these intriguing results, the existence of an effect of COMT variation on pain sensitivity is still strongly debated, as some subsequent behavioral studies using larger sample size have failed to show a substantial association (e.g. [13]–[17]). In the last few years, evidence produced by several groups has suggested that the effect of COMT polymorphism on pain sensitivity is generally not observed for the initial pain provocations, but rather becomes apparent in later phases of a testing session [8], [18], [19]. Thus, it is possible that the inconsistency in the literature on the effects of COMT is attributable to the delayed onset of this effect, which some studies might have failed to capture.

The aim of the present study was to test the hypothesis that the effect of COMT on pain modulation emerges in the setting of a repeated pain challenge, as proposed by Jensen and colleagues [19]. In order to test our hypothesis we reanalyzed the fMRI activations in response to early and late stimuli in a series of repeated heat pain stimulations, using data from three previous experiments from our laboratory [20]–[22].

## Methods

In the present study, we pooled data from three experiments [20]–[22], to obtain a total of 54 healthy normal right-handed subjects (see Table 1 for demographic information). Although the original experiments were not specifically designed to test the hypothesis evaluated in the present work (but rather to investigate the brain mechanisms of placebo, nocebo and acupuncture analgesia), all of them included two, completely identical, fMRI runs ‘at baseline’ (i.e., in the absence of any experimental treatment) at the beginning of the visit (see below). The data from these baseline runs were here pooled and reanalyzed. Additional details on the experimental procedures which are specific to each of the three studies, but irrelevant for the present manuscript, will not be further discussed. Ethical approval for the present study was obtained from Massachusetts General Hospital's Institutional Review Board and the experiments were performed in accordance with the Helsinki Declaration of human research ethics. All subjects gave written informed consent.

**Table 1:** Descriptive statistics for the three genotype groups studied.

COMT val^158^met genotype	N	Age (mean ±SD)	Ethnicity %
Met/Met	12 (66.7% F)	28.3±6.8	58.3% white 33.3% Asian 8.3% black
Val/Met	22 (45% F)	25.5±2.8	72.7% white 13.6% Asian 9.1% black 4.5% hispanic
Val/Val	20 (60% F)	25.7±4.8	70% white 15% Asian 10% black 5% mixed

All subjects participated in two behavioral testing sessions and one fMRI scanning session. The two behavioral sessions were aimed at familiarizing subjects with the rating scales, determining appropriate stimulus intensities (i.e., the temperatures eliciting subjective intensity ratings in the LOW pain range, ∼5/20, and HIGH pain range, ∼15/20), and assessing the stability of the subjective ratings; for more details see [23].

During the fMRI session, subjects received two identical pseudorandom sequences of calibrated heat pain stimuli (4 LOW and 4 HIGH) one each during a ∼6 minute scan acquisition (run), on the right volar forearm. Between the two runs, there was a minute pause, during which a member of staff moved the thermode from the ulnar side to the radial side of the arm (or vice versa; counterbalanced), to avoid sensitization of the skin. There was no other task between run 1 and run 2. The temperatures of the LOW and HIGH pain stimuli were kept constant during both functional runs included in this study. Heat stimuli were delivered using a TSA-2001 Thermal Sensory Analyzer with a 3 cm×3 cm probe (Medoc Advanced Medical Systems, Rimat Yishai, Israel) running the COVAS software. All stimuli lasted 12 seconds, including 2.5 second to ramp up towards the target temperature and to ramp down to baseline (32°C). The inter-stimulus interval ranged from 24 to 30 seconds. The onset of the stimuli was signaled by a change in color of the fixation cross (black during rest, red during stimulation). After a delay of 4–8 seconds from stimulus offset, subjects used a button box to rate pain intensity on a digitized version of the Gracely Sensory scale [24].

At the end of the first behavioral session, all subjects meeting criteria for continuation in the study had two 10 ml tubes of blood drawn for genetic analyses. Blood collected was sent in two Acid Citrate Dextrose (ACD) solution A tubes to the Harvard Genotyping core facility. Genomic deoxyribonucleic acid (DNA) was extracted and quantified using well established methods [25]. The DNA was used to determine the individual COMT genotypes of the subjects by direct resequencing using ABI capillary platform (ABI 3730xl). The polymerase chain reaction (PCR) was used to amplify the region of the COMT gene that contains the valine to methionine polymorphism at nucleotide 158. The sequences of the oligonucleotide primers used for the PCR were 5′-TCA CCA TCG AGA TCA ACC CC-3′ and 5′-GAA CGT GGT TGT AAC ACC TG-3′ [26]. Both strands of PCR products were sequenced using the standard resequencing protocol (Current Protocols in Human Genetics v.2, unit 7.9, October 2008). Sequence reads were analyzed by PolyPhred. Mutations were scored on both strands.

The General Linear Model (GLM), including the between-subject factor Genotype (levels: ‘VAL/VAL’, ‘VAL/MET’ and ‘MET/MET’) and the within-subject factor Stimulus Level (‘LOW’, ‘HIGH’), was first used to assess whether the three genotype groups exhibited significantly different pain sensitivity on average. The subjective pain ratings, averaged by stimulus level, and the individually tailored temperatures were used as dependent variables in separate analyses. Based on the consistently reported observation that the effect of COMT polymorphism on pain sensitivity is generally not observed for the initial pain provocations, but rather becomes apparent in later phases of a testing session [8], [18], [19], we also analyzed the two runs separately, to parallel our imaging analyses (see below). All statistical analyses of behavioral data were performed using the statistical software SPSS for Windows, version 16.0.

Brain imaging was performed with a 3-axis gradient head coil in a 3 Tesla Siemens MRI System (Allegra/Trio) equipped for echo planar imaging. The scanning parameters were identical for all subjects. Thirty axial interleaved slices (4 mm thick with 1 mm skip) parallel to the anterior and posterior commissure covering the whole brain were acquired with TR = 2000 ms, TE = 40 ms, flip angle = 90°, and a 3.13×3.13 mm in-plane spatial resolution. A high-resolution 3D MPRAGE volume for anatomical localization was also collected.

fMRI data processing was carried out using FEAT (FMRI Expert Analysis Tool) Version 5.98, part of FSL (FMRIB's Software Library, www.fmrib.ox.ac.uk/fsl). The following pre-statistics processing was applied: fieldmap-based EPI unwarping using PRELUDE+FUGUE, non-brain removal using BET; spatial smoothing using (FWHM = 5 mm); grand-mean intensity normalisation by a single multiplicative factor, and high-pass temporal filtering (sigma = 59.0 s). Time-series statistical analysis was carried out using FILM with local autocorrelation correction. For each subject, the following contrasts were computed: ‘LOW pain vs. baseline’, ‘HIGH pain vs. baseline’ and ‘HIGH vs. LOW pain’. As we did for the psychophysical ratings, in order to unveil potential genotype effects emerging only in later phase of the testing session (see above), we also performed these analyses for each run separately. Registration to high resolution structural and standard space images was carried out using FLIRT. Group level analyses were carried out to compare brain responses to LOW and HIGH pain stimuli across genotypes using FLAME (FMRIB's Local Analysis of Mixed Effects) stage 1. In accordance with the observation that val/val and met/met homozygotes are characterized by the strongest and weakest COMT enzymatic activity respectively (while val/met exhibit intermediate activity [11], [12]), a direct comparison was performed between the two homozygote groups. Even though the met/met and the val/val group participants were perfectly balanced across scanners (an equal number of subjects within each group were scanned with the Trio and Allegra scanners), and the gender distribution was also well balanced (female subjects represented 66.7% and 60% of the met/met and val/val group respectively), gender and scanner type were included as covariates of no interest in the design matrix. Z (Gaussianised T/F) statistic images were thresholded using clusters determined by Z>1.96 and a (corrected) cluster significance threshold of P = 0.05. In order to further explore our data, we also performed paired t-tests, in which the brain responses to the pain stimuli in the two runs were directly compared within-subject, using the same criteria for significance adopted for the between-subject analyses. The statistically significant clusters from the between-subject analyses were masked with the results of these within-subject analyses, in order to assess whether any potential group differences emerging in the second run might be driven by an increase in BOLD signal in one homozygote group, a decrease in the other, or both.

Finally, the values of the pain-related percent change in BOLD signal for all three groups were extracted from the activation peaks identified in the GLM analyses using FSL's Featquery tool, and then plotted with Statistica 10.0 (StatSoft, Inc). Human brain atlases were used for anatomical reference for the forebrain [27] and the brainstem [28].

## Results

Of the 54 healthy subjects included in this study, 20 (∼37%) were found to be homozygous for the ^158^val allele, 12 (∼22%) homozygous for the ^158^met allele, and 22 (∼41%) heterozygous. See Table 1 for descriptive statistics of the three groups. No significant age differences were observed among the three groups, F(2,51) = 1.493, p>0.05. Among both the met/met and the val/val individuals, 50% were scanned with the Allegra scanner and 50% were scanned with the Trio TIM scanner. Among the heterozygous, 64% were scanned with a Trio TIM scanner, and the remaining 36% were scanned with an Allegra scanner.

Due to the subjective calibration of heat stimuli in all subjects, we did not detect any significant group differences in the evoked pain ratings (main effect of GENOTYPE: F(2, 52) = 0.67, p = 0.51, n.s.; GENOTYPE * STIMULUS RATING interaction: F(2,52) = 2.15, p = 0.13, n.s.). No differences were observed even when separate analyses were performed for each of the two runs independently.

Furthermore, we did not observe any group differences in the temperatures individually calibrated to evoke the target pain levels either (main effect of GENOTYPE: F(2, 52) = 1.71, p = .19, n.s.; GENOTYPE * STIMULUS LEVEL interaction: F(2,52) = 0.10, p = 0.90, n.s.). The temperatures needed to evoke LOW pain (SD) in met/met, val/met and val/val were 45.7 (0.9), 45.9 (1.5) and 45.3 (1.7), respectively; those needed to evoke HIGH pain were 48.5 (0.8), 48.8 (1.3) and 48.2 (1.3), respectively.

**Figure 1:**
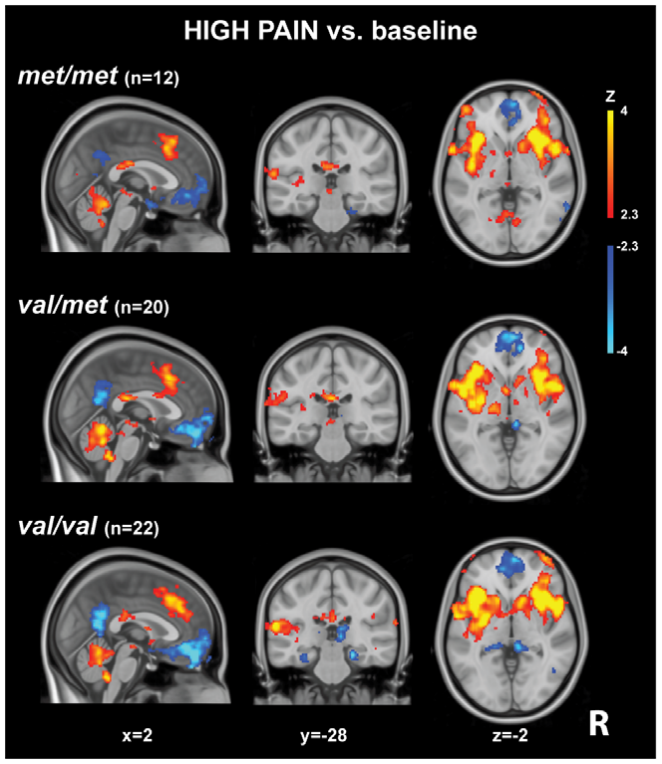
HIGH-pain related brain activations, overlaid on the MNI152 standard brain.

As previously reported [23], the application of pain stimuli in the total cohort evoked stimulus intensity dependent fMRI signal increases in regions commonly activated in response to experimentally applied heat pain, including contralateral (left) primary somatosensory cortex (S1) and primary motor cortex (M1), bilateral anterior/middle cingulate cortex, supplementary motor area, insula, superior and inferior parietal lobules, secondary somatosensory cortex (S2), frontal poles, occipital cortex, thalamus, putamen, periaqueductal gray (PAG), medulla and cerebellum, which is consistent with published reports [29]–[31]. Figure 1 shows the activations and deactivations evoked by HIGH pain in the three groups for illustrative purposes.

**Figure 2:**
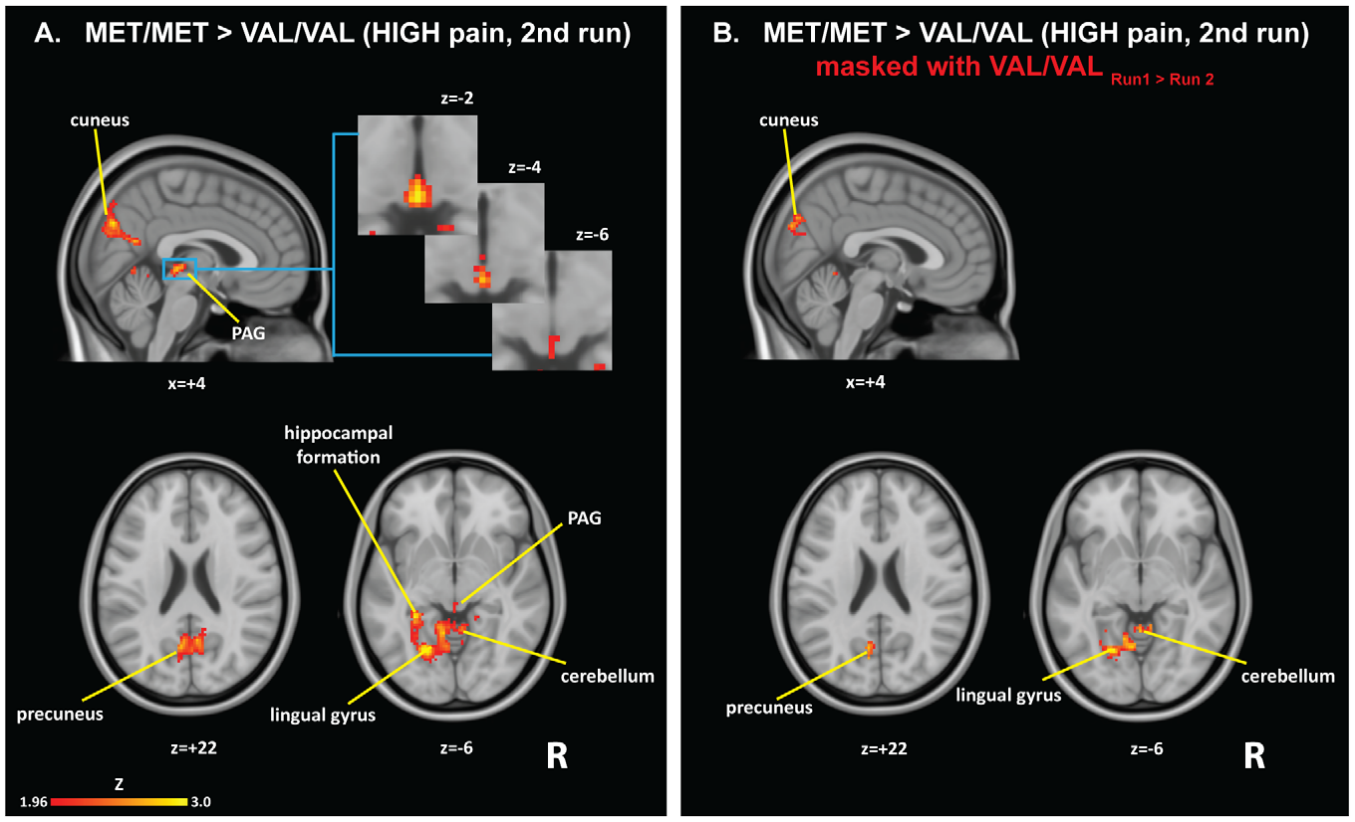
Genotype effects on pain-evoked brain activations. A. Genotype effect on pain related activations, emerging for HIGH pain in the second run. Brain activations for the contrast met/met>val/val, overlaid on the MNI152 standard brain. Right side = right hemisphere. B. Significant activations from the between-subject analyses masked with regions in which val/val exhibited a reduction in BOLD signal in Run 2 as compared to Run 1.

When comparing the pain-evoked brain activations across groups, no genotype differences were observed when the two runs were combined. However, when separate analyses were performed for the first and second run, a significant genotype effect emerged. In the second run, the met/met subjects, compared to val/val subjects, exhibited higher BOLD signal in response to HIGH pain (but not to LOW pain) in a number of cortical and subcortical structures, including the periaqueductal gray matter (PAG), hippocampal formation, lingual gyrus, calcarine cortex, precuneus, cuneus, superior and middle occipital gyri and cerebellum (p<0.001, cluster corrected; Table 2 and Fig. 2). No brain regions showed higher pain-related BOLD signal in the val/val individuals. Results were very similar when the analysis was run without including gender and scanner type as covariates.

**Table 2:** Brain structures exhibiting a significant genotype effect for HIGH pain in the second run.

*Side*	*Label (peak location in italic)*	*N_voxels_*	Zstat_max_	x_max_	y_max_	z_max_
L	*Cerebellum (vermis)*, lingual g., HF, calcarine cx	163	3.11	−6	−56	−2
L	Lingual g.	109	3.16	−18	−64	−8
L	*Lingual g.*, HF	105	3.69	−24	−50	−2
L+R	Precuneus	91	3.16	8	−58	20
R	*Precuneus*, cuneus	53	2.87	4	−76	34
L	Calcarine cx	45	2.77	−16	−70	10
L	Occipital pole	40	2.86	−18	−90	8
R	*PAG*	32	3.01	2	−28	−2
R	Precuneus	20	2.6	12	−74	52

For descriptive purposes, the significant cluster was partitioned in subclusters by further applying a voxelwise threshold of z = 2.3. Subclusters with a N_voxels_≥20 are described.

Abbreviations: cx = cortex, g. = gyrus, HF = hippocampal formation, PAG = periaqueductal gray.

**Figure 3:**
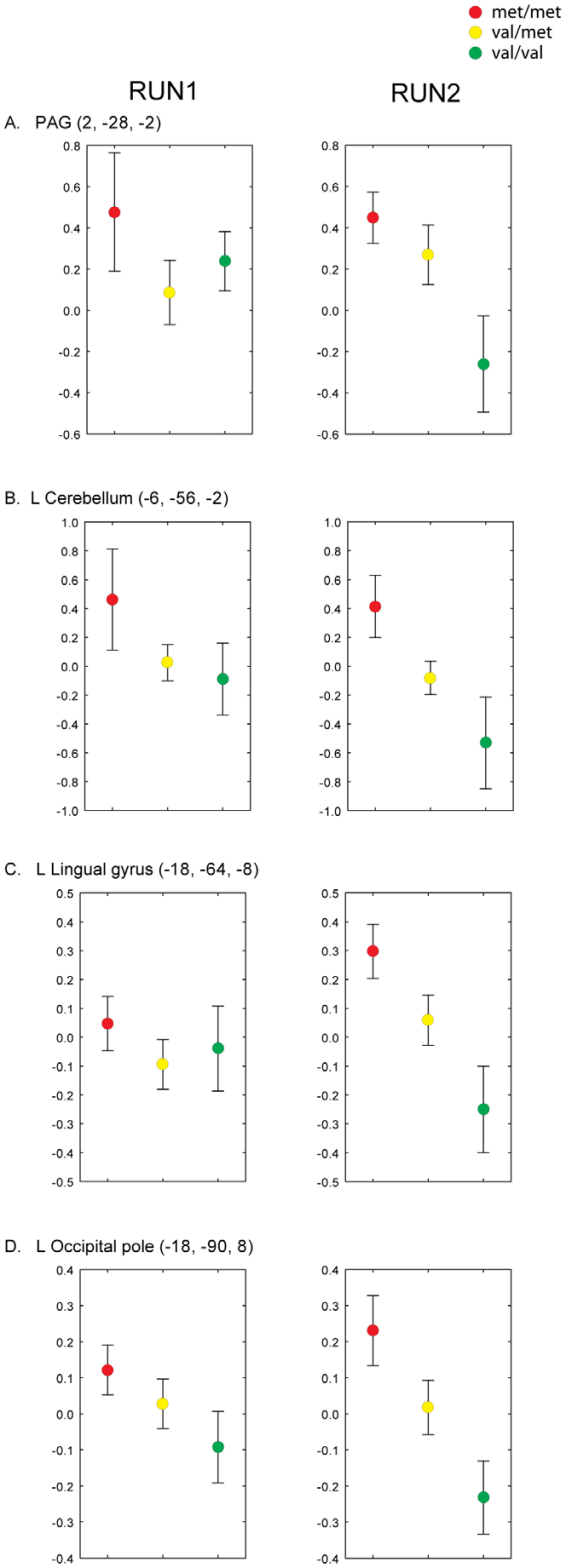
HIGH Pain-related percent signal change in representative brain regions across three groups in both run 1 and run 2. Bars represent mean ± SEM.

In order to assess whether the group differences observed in Run 2 were driven by a within-session increase in BOLD signal in one homozygote group, a decrease in the other, or both, the pain-evoked brain responses observed in the two runs (run 1 versus run 2) were directly compared using within-subject paired analyses, within the regions that exhibited statistically significant genotype differences in the between subject analyses. These analyses revealed that the val/val subjects exhibited a lower BOLD signal in run 2 compared to run 1, within the majority of the areas showing a genotype effect in the between subject analyses, including lingual gyrus, calcarine cortex, precuneus, cuneus, superior and middle occipital gyri and cerebellum (p<0.001, cluster corrected). No across-run changes were observed in the other groups (i.e. met/met and val/met groups). While these within-subject analyses did not reveal a significant cluster for the PAG, the examination of the % BOLD signal change extracted from this region (Figure 3) reveals a pattern which is in line with the paired analyses, i.e., the val/val subjects exhibit lower BOLD signal in run 2, compared to run 1. In general, Figure 3 indicates that a gene dosage effect on the HIGH pain-related activations, with the heterozygous subjects exhibiting intermediate BOLD signals between the two homozygous groups, appeared in the second run within several regions. While this effect was generally driven by a BOLD signal reduction in the val/val subjects (Figure 3, panels A–B), in some cases it was also driven by a concomitant increase in the BOLD signal in the met/met subjects (Figure 3, panels C, D). When the HIGH versus LOW pain contrast was compared across genotypes, we observed that the met/met subjects exhibited activations of larger magnitude compared to the val/val subjects in the first run, in regions of the occipital cortex and a small portion of the posterior cingulate cortex/precuneus (p<0.05, cluster corrected). These regions exhibited very little overlap with the areas where the main genotype effect on the HIGH versus baseline contrast was observed in run 2. No other differences were observed. A summary of the results from all contrasts is provided in Table 3.

**Table 3:** Summary table of the results from all contrasts.

*Between subject analyses*	*met/met>val/val*	*val/val>met/met*
Run 1 LOW	**n.s.**	**n.s.**
Run 1 HIGH	**n.s.**	**n.s.**
Run 1 HIGH vs LOW	**occipital gyri, calcarine cx, posterior cingulate cx/precuneus**	**n.s.**
Run 2 LOW	**n.s.**	**n.s**
Run 2 HIGH	**PAG, HF, lingual g., calcarine cx, precuneus, cuneus, superior and middle occipital gyri, cerebellum**	**n.s**
Run 2 HIGH vs LOW	**n.s**	**n.s**

*Within subject analyses*	*met/met*	*val/val*
Run 1>Run 2 HIGH	**n.s.**	**lingual g., calcarine cx, precuneus, cuneus, superior and middle occipital g., cerebellum**
Run 2>Run 1 HIGH	**n.s.**	**n.s.**

Abbreviations: see Table 2 caption.

## Discussion

In the present study, we evaluated the fMRI responses to experimental pain stimuli in subjects genotyped for the COMT val^158^met polymorphism. Our results demonstrate that individuals with the met/met genotype exhibit stronger pain-evoked BOLD signals in a number of cortical and subcortical structures (PAG, hippocampal formation, lingual gyrus, calcarine cortex, precuneus, cuneus, superior and middle occipital gyri and cerebellum), in spite of identical pain ratings between genotype groups. Interestingly, this effect emerges only after repeated noxious stimulation. This observation is consistent with the results from previous studies showing that the COMT val^158^met polymorphism exhibits a detectable effect on measures of pain sensitivity (e.g., verbal ratings, or the stimulus intensity required to achieve a target pain level) only after repeated or prolonged stimulation [8], [18], [19]. Thus, while the experiments from which the present data were pooled were not originally designed for this purpose, our results further corroborate the hypothesis, proposed by Jensen and colleagues [19], that the influence of COMT val^158^met on central pain processing becomes apparent only when the pain system is repeatedly and robustly challenged. More specifically, an examination of the results of the within-subject analyses (Figure 2b), as well as a comparison of the % signal change evoked by pain in the two runs (Figure 3), suggests that the differences appear to be primarily driven in several regions by a BOLD signal reduction in the val/val subjects (Figure 3, panels A–B). This observation indicates that repeated pain stimulation is accompanied by habituation in the activity within these structures in the val/val subjects but not in the met/met subjects. Since the difference in the adaptation profile is not accompanied by differences in behavioral measures, we speculate that adaptation to repeated pain stimulation might occur at earlier stages of neural processing, for instance at the spinal cord level, in the val/val subjects, whereas in met/met subjects supraspinal mechanisms would have to be recruited in a more sustained fashion to counteract a possible hypofunctionality of these more peripheral mechanisms. Future studies will need to assess this hypothesis.

In some regions, however, the group effect was also driven by an increase in the BOLD signal in the met/met subjects; e.g., occipital pole and the lingual gyrus/posterior cingulum/precuneus region (Figure 3, panels C, D). Increased activation of the posterior cingulum/precuneus region has previously been implicated in pain modulation [32]–[33] and it is possible that the increased posterior cingulate/precuneus BOLD signal in met/met subjects during the first run reflects higher involvement of a cortical pain modulatory network. However, as the role of the regions presenting these patterns in pain processing have been heavily underexplored to date, any attempt of explaining the functional significance of these results is purely speculative at this time. At the very least, our results do demonstrate that the COMT–related impact on pain-evoked brain activity appears to be complex and region-specific.

Even though we observed a genotype effect on brain activation, no measures of pain sensitivity were different across groups. In fact, the temperatures capable of eliciting the calibrated LOW and HIGH pain stimuli were similar, and the ratings (which were similar at the beginning of the experiment due to the successful calibration of the heat intensities) did not diverge in the last part of the experiment. This is in agreement with an increasing body of literature in which conflicting findings have emerged for COMT (and, more in general, most of the identified “pain genes”; [6]). While some studies do report a significant effect of COMT on sensitivity to experimental pain, several others have only showed a week association e.g. [34], [35], or no association e.g. [13]–[17]. Similar inconsistencies are found in the clinical literature where some studies show an effect of COMT genotype on reports of clinical pain, or vulnerability to developing chronic pain conditions [36]–[40], but several others report the lack of a substantial association [41], [42].

The presence or absence of an association between a particular gene and a pain phenotype may be highly sensitive to a variety of factors. For instance, an association between COMT variation and pain responses was found to be significant only for thermal pain, not for ischemic and mechanical pain [18], suggesting that COMT might have different effects on different pain modalities. However, this explanation alone cannot fully account for the inconsistencies encountered in the literature, as other studies using heat stimuli have failed to report an effect of COMT on pain (e.g., [15]). Our observation that the brain responses to pain in subjects with different COMT genotypes start diverging only after repeated pain stimulations suggests that the time profile and/or the cumulative intensity of the noxious stimulation might explain why an effect is observed in some studies and not in others. A possible clinical implication of this observation could be that COMT genotypes might have their strongest impact on the long-term probability of developing intermittent/episodic pain conditions. In a more recent study investigating event related potentials in patients with low back pain and healthy controls [43], the authors found that the met allele was associated with augmented cortical processing of experimental pain in patients but not in controls, supporting the notion that COMT is more important in individuals with already heightened pain sensitization.

Furthermore, our data show that in both runs met/met subjects exhibit similar BOLD signal in the periaqueductal gray (PAG), a key structure within the descending pain modulatory system [44], [45], whereas in the val/val subjects this response is reduced in the second run. In a previous neuroimaging study, we demonstrated that the PAG is functionally connected to important pain regulatory brain regions, such as the ACC and the rostral ventromedial medulla [46]. We suggest that the more sustained recruitment of PAG in met/met individuals might represent a compensatory mechanism counteracting the lower neuronal levels of enkephalin, which have been shown to be associated with chronic activation of the dopaminergic system [47]–[49]. Thus, met/met subjects might develop compensatory mechanisms counteracting their heightened sensitivity and vulnerability, which could reduce the likelihood of detecting an effect of COMT on behavioral pain sensitivity measures. However, the mobilization of these mechanisms might be dependent on a variety of factors (including pain modality tested, subjects' demographics, type of disease, etc), which would explain why a COMT effect on pain is observed in some studies and not in others. For instance, COMT related differences might be most readily detected in the setting of a strong challenge to the inhibitory system, such as among some chronic pain patients.

Finally, our results suggest that the effect of COMT on pain might be, albeit genuine, too small to be reliably measured using verbal ratings. In some cases, this effect might be more consistently observed at the brain activity level, since brain activity measures might be considered more proximal (‘intermediate’) phenotypes to perception than subjective reports. In line with this hypothesis is a recent fMRI study [50], which reported stronger activations within regions of the ‘pain matrix’ (particularly in the posterior ACC/mid-cingulate cortex) in met/met subjects (compared to val/met and val/val combined), despite the absence of a difference in pain ratings. On the other hand, the lack of differences in pain ratings in the presence of different patterns of brain activation could also suggest that the COMT genotype might not affect ‘pain processing’ per se, but other brain functions. In fact, as a result of its modulatory role on widespread neuroendocrine and neurotransmitter systems (dopamine, norepinephrine, epinephrine), variation in COMT has been shown to affect brain activity in a wide variety of domains, including attention, working memory and affective regulation [51], rendering unlikely that pain processing and perception would be specifically targeted [6]. Another possibility, which future studies will need to address, is that COMT might affect neurovascular coupling.

In conclusion, COMT appears to affect brain responses to experimentally induced pain, and this effect reveals itself in the context of repeated painful stimulations. However, given our relatively small sample size and unbalanced group Ns, larger and more balanced studies will need to be conducted in order to confirm the validity and generalizability of our observations. Furthermore, future experiments will also need to be specifically designed to test the hypothesis here proposed that the met/met subjects might develop compensatory mechanisms counteracting their putative heightened sensitivity to pain.
